# Hederagenin regulates the migration and invasion of hepatocellular carcinoma cells through FOXO signaling pathway

**DOI:** 10.1371/journal.pone.0310930

**Published:** 2024-10-09

**Authors:** Shuchang Bao, Songzhe Li, Yang Sun

**Affiliations:** College of Basic Medicine, Heilongjiang University of Chinese Medicine, Harbin, China; Shantou University Medical College, CHINA

## Abstract

**Objective:**

This study aimed to elucidate the effects of Hederagenin (HG) on hepatocellular carcinoma (HCC) and explore its potential molecular mechanisms.

**Materials and methods:**

Virtual screening was employed to identify potential targets within core pathways of liver cancer and to analyze the possible mechanisms of HG. CCK-8 assays were used to assess the viability of HCC cells, while Hoechst 33342/PI staining was utilized to evaluate apoptosis. The migration and invasion abilities of HCC cells were examined using Transwell and scratch assays, and single-cell cloning ability was assessed via colony formation assays. Subsequent qRT-PCR was conducted to determine the mRNA expression levels of FOXO1 and FOXO6 following HG treatment. Western blot (WB) analysis was employed to measure the protein expression levels of IGF1R, FOXO1, FOXO6, MMP2, MMP9, and VEGFA, as well as the phosphorylation status of FOXO1 Ser249.

**Results:**

Virtual screening indicated that HG might exert antitumor effects through the FOXO signaling pathway. Experimental results demonstrated that HG induces apoptosis in a dose-dependent manner and inhibits the proliferation, migration, invasion, and single-cell cloning ability of HCC cells. After HG treatment, FOXO1 expression was upregulated, while the expression levels of IGF1R, phosphorylated FOXO1 Ser249, MMP2, MMP9, and VEGFA were downregulated.

**Conclusion:**

In summary, our study is the first to demonstrate that HG regulates the phosphorylation of FOXO1, affecting the proliferation, migration, and invasion of HCC cells. The findings suggest that HG can inhibit the migration of HCC cells in vitro. The data indicate that HG-mediated targeting of the FOXO1/FOXO6 pathway holds promise as a novel therapeutic approach.

## 1 Introduction

Primary liver cancer is a common malignancy, with hepatocellular carcinoma (HCC) accounting for 70%-80% of cases [1]. Despite advancements in the treatment of early-stage liver cancer, effective therapeutic strategies for patients with advanced stages remain lacking. According to data from the Surveillance, Epidemiology, and End Results (SEER) program of the National Cancer Institute, the average five-year survival rate for HCC patients in the United States is less than 20%, with the survival rate for those with advanced metastatic disease potentially as low as 2.5% [2]. The low survival rate is not only due to late diagnosis and the limited effectiveness of surgical interventions but also to the high recurrence rate and the aggressive migration and invasion of liver cancer cells. For patients with advanced-stage disease, tumors often exhibit distant metastasis and invasion, limiting surgical options to isolated tumors without vascular invasion [2]. It is estimated that approximately 90% of cancer-related deaths result from the growth of metastatic tumors in locations other than the primary site [3], a process closely linked to the regulation of Forkhead box O (FOXO) proteins.

The FOXO family has been shown to mediate energy metabolism and tumorigenesis by specifically activating coordinated transcription programs. Dysregulation of FOXO functions can lead to uncontrolled cell proliferation and impaired DNA damage repair, contributing to cancer development. Upon phosphorylation by overexpressed AKT, FOXO6, which lacks a nuclear export sequence (NES), remains localized in the nucleus and functions independently of external signaling [4]. Other FOXO family members are excluded from the nucleus and degraded via ubiquitination. AKT is positively regulated by upstream targets such as insulin-like growth factor 1 (IGF1). The binding of IGF1 to its receptor, IGF1R, directly activates the PI3K/Akt pathway and inhibits the transcriptional activity of the tumor suppressor FOXO1, which may result in poor prognosis [5]. The role of FOXO6 in tumors is currently controversial, suggesting potential research value. Some studies, such as those by Hu et al., have shown that upregulation of FOXO6 can inhibit the proliferation of lung cancer cells [6]. Conversely, Yu et al. demonstrated that downregulation of FOXO6, inhibiting the activation of the PI3K/Akt signaling pathway, can suppress the proliferation, invasion, and glycolysis of HCC cells [7].

Hederagenin (HG), a pentacyclic triterpenoid compound, is widely distributed in various medicinal plants [8]. It has been shown to possess broad potential antitumor effects in both in vitro and in vivo studies [9–11]. Mechanistically, HG is closely associated with several signaling pathways, including nuclear factor-κB (NF-κB), Janus kinase/signal transducers and activators of transcription (JAK/STAT), and intracellular phosphoinositide 3-kinase (PI3K)/protein kinase B (AKT). By modulating these pathways, HG influences apoptosis, autophagy, and cell cycle progression, thereby impacting tumor development, reducing drug resistance, and exerting anti-inflammatory and antitumor effects[12–16]. To date, no studies have investigated the effects of HG on the migration and invasion of liver cancer. Given that FOXO is a critical downstream transcription factor in the PI3K/AKT pathway, we hypothesized that HG might inhibit the migration and invasion of liver cancer cells through the FOXO signaling pathway.

This study explored the effects of HG on inhibiting the migration and invasion of HCC cells by regulating the phosphorylation of FOXO1 and FOXO6. Our findings reveal that HG inhibits IGF1R, upregulates FOXO1, and downregulates FOXO6, as well as p-FOXO1 Ser249, MMP9, MMP2, and VEGF expression.

## 2 Materials and methods

### 2.1 Virtual screening of liver cancer pathway

#### 2.1.1 Collection of potential targets

The ’ Invasion of liver cancer ’ was used as the key word to mine the potential targets involved in liver cancer invasion in the GeneCards database (https://www.genecards.org) (downloaded in November, 2023). The collected targets only retained information with a correlation score ≥ 50.

#### 2.1.2 Gene enrichment analysis

The targets obtained in 1.1.1 were entered into the Metascape database (http://metascape.org) [17,18], with a statistical significance threshold set at Pvalue< 0.01. The analytic mode was tailored, and then, the Kyoto Encyclopedia of Genes and Genomes (KEGG) pathway analysis was conducted. The GO gene function enrichment analysis is conducted at the levels of Molecular function (MF), Biological process (BP), and Cellular component (CC).

### 2.2 TCGA database target expression prediction

#### 2.2.1 Screening of HCC genes in TCGA database

The screening samples were obtained from The Cancer Genome Atlas (TCGA) database (https://portal.gdc.cancer.gov/). The screening of the disease was restricted to TCGA-LIHC, irrespective of gender, survival status, and mortality. A total of 424 STAR-Counts files were acquired, comprising 50 samples from healthy individuals and 374 samples from those with diseases. The R4.2.1 version of the ’limma’ package was utilized to standardize the values into FPKM values.

#### 2.2.2 Gene difference analysis

The expression profile difference analysis was conducted utilizing the ’limma’, ’ggplot2’, and ’ggpubr’ packages. Initially, the data samples of liver cancer were inputted, followed by the input of the standardized name of the target gene to be screened. This was done to compare the variations in gene expression between normal samples and diseased samples. A significance level of *P* < 0.001 was employed as the screening criterion and denoted as ’ * * * ’.

### 2.3 Molecular docking verification

#### 2.3.1 Collection of hederagenin and docking targets

The mol2 file of HG is sourced from the ZINC20 database[19]. The protein crystal structure of the docking target was obtained from the RCSB PDB database (http://www.rcsb.org/). The search criteria used were ’Homo sapiens’ and ’Protein’, and the PDB number was recorded after downloading.

#### 2.3.2 Molecular docking software and docking scheme

The vina module in autodockTools 1.5.6 is utilized, much like in the previous study [20,21]. The prediction of HG small molecules and target proteins involved the removal of water molecules, hydrogenation pretreatment, semi-flexible docking, and blind docking approaches. The default values were used for the other parameters. The docking results are determined by selecting the binding energy with the lowest value and then displayed visually.

### 2.4 Materials and methods

#### 2.4.1 Chemicals

The HG compound was acquired from Solarbio (Beijing, China, SH8030) with a purity≥ 98%. It was diluted in dimethyl sulfoxide (DMSO) and stored at a temperature of -20°C. Prior to the experiment, the DMEM medium was utilized to dilute to the necessary concentration.

#### 2.4.2 Cell culture

The human hepatocellular carcinoma cell line Huh-7 was purchased from Haixing Biosciences (Suzhou, China), and the SMMC-7721 cell line was obtained from FuHeng BioLogy (Shanghai, China). All cells were cultured in Dulbecco’s Modified Eagle Medium (DMEM) (BOSTER, Wuhan, China, PYG0101). The complete DMEM medium included 10% fetal bovine serum (FBS) and 1% antibiotic solution containing 100 U/ml penicillin and 100 μg/ml streptomycin. Cells were maintained in a humidified atmosphere of 5% CO_2_ at 37°C.

#### 2.4.3 Cell viability assay

Huh-7 and SMMC-7721 cells were seeded at a density of 2.5 × 10^3^ cells per well in 96-well plates and co-cultured with specific concentrations of HG for 24 hours at 37°C. Cell viability was assessed using the Cell Counting Kit-8 (CCK-8) assay (MeilunBio, Dalian, China, MA0218-2-Oct-28G) following the manufacturer’s instructions. After adding 10 μL of CCK-8 solution to each well, the plates were incubated in a cell culture incubator for 2 hours, and the optical density at 450 nm was measured using a microplate reader (Tecan Infinite 200 PRO, Switzerland). The IC50 values were calculated using the Four Parameter model on the IC50 calculator website.

#### 2.4.4 Apoptosis staining experiment

Huh-7 and SMMC-7721 cells were seeded at a density of 1 × 10⁴ cells per well in 24-well plates and co-cultured with specific concentrations of HG for 24 hours at 37°C. Following incubation, Hoechst 33342 solution (MeilunBio, Dalian, China, MA0126) and propidium iodide (PI) solution (Sigma, Shanghai, China, P4170) were added. The cells were then observed under a fluorescence microscope (EVOS, USA), and images were captured within 1 hour.

#### 2.4.5 Cell migration and invasion assay

In the migration assay, treated Huh-7 and SMMC-7721 cells (2.5 × 10⁴ cells/well) were seeded into the upper chamber of a Transwell insert (Corning, USA, 3422). For the invasion assay, a thin-layer gel method was employed, where Matrigel (Corning, USA, 356234) diluted with serum-free medium was applied to the upper chamber of the Transwell insert and allowed to solidify. Subsequently, treated Huh-7 and SMMC-7721 cells (4.5 × 10⁴ cells/well) were added to the upper chamber containing Matrigel. The lower chamber was filled with DMEM medium containing 10% fetal bovine serum, and the cells were treated with HG for 24 hours. After 24 hours, cells on the upper surface of the polycarbonate membrane were removed using a sterile cotton swab. The remaining cells were fixed with 4% paraformaldehyde (Biosharp, Anhui, China, BL539A) for 15 minutes and stained with 0.1% crystal violet (ZhiYuan, Tianjin, China, Q/12HG 5690–2002) for 20 minutes. The membrane was then air-dried, and the migrated and invaded cells were counted under an EVOS optical microscope in three randomly selected fields of view.

#### 2.4.6 Cell scratch test

Huh-7 and SMMC-7721 cells (2 × 10⁵ cells/well) were seeded into 6-well plates and cultured at 37°C until they reached 100% confluence. The cells were then cultured in serum-free medium for 24 hours. A sterile toothpick was used to create vertical scratches in each well, after which the cells were co-cultured with specific concentrations of HG for an additional 24 hours. The scratch gaps at 0 hours and 24 hours were photographed using an EVOS optical microscope.

#### 2.4.7 Plate cloning experiment

Huh-7 and SMMC-7721 cells (7.5 × 10^2^ cells/well) were seeded into 6-well plates and co-cultured with specific concentrations of HG. The culture was stopped when the majority of cells had formed colonies. The cells were then fixed with 4% paraformaldehyde for 20 minutes and stained with 0.1% crystal violet for 5 minutes. After washing three times with PBS, the plates were air-dried. The colonies were photographed using a camera and an EVOS optical microscope.

#### 2.4.8 RNA extraction and Quantitative Real-time PCR (qRT-PCR) analysis

After co-culturing Huh-7 and SMMC-7721 cells with the specified concentration of HG for 24 hours, total RNA was extracted according to the instructions provided with the RNAeasy™ Animal RNA Extraction Kit (Centrifugal Column) (Beyotime, Shanghai, China, R0027). The RNA was then reverse transcribed into cDNA using the BeyoRT™ III First Strand cDNA Synthesis Master Mix (5X) (Beyotime, Shanghai, China, D7182M). The thermal cycling conditions for cDNA synthesis were set at 42°C for 10 minutes, followed by 80°C for 10 minutes. According to the manufacturer’s instructions, PCR amplification of the cDNA was performed using ChamQ Universal SYBR qPCR Master Mix (Vazyme, Nanjing, China, Q711-02). GAPDH mRNA was used as an internal control, and the relative expression levels of IGF1R, FOXO1, FOXO6, MMP2, MMP9, and VEGFA (primer sequences are listed in Table 1) were calculated using the 2-ΔΔCt method. The primers were synthesized by Comate Bioscience Co., Ltd. (Jilin, China). The thermal cycling conditions were as follows: initial denaturation at 95°C for 10 minutes, followed by 40 cycles of 95°C for 30 seconds, 56°C for 30 seconds, and 72°C for 45 seconds. The melting curve analysis was performed with the following conditions: 95°C for 15 seconds, 60°C for 1 minute, and 95°C for 15 seconds, for a total of one cycle.

**Table 1:** qRT-PCR primer sequences.

Genes	Sequences
GAPDH	F:5’- TGGTATCGTGGAAGGACTCA-3’
	R:5’- CCAGTAGAGGCAGGGGATGAT-3’
FOXO1	F:5’- AGGATAAGGGTGACAGCAACAG-3’
	R:5’- TTGCTGTGTAGGGACAGATTATGAC-3’
FOXO6	F:5’- AGAGTTCGTGGTGGATGCTG-3’
	R:5’- GCTTCTTCTTGCTCGCCTTG-3’
IGF1R	F:5’- TCGACATCCGCAACGACTATC-3’
	R:5’- CCAGGGCGTAGTTGTAGAAGAG-3’
MMP2	F:5’- GATACCCCTTTGACGGTAAGGA-3’

#### 2.4.9 Protein preparation and Western Blot (WB)

After co-culturing Huh-7 and SMMC-7721 cells with a specific concentration of HG for 24 hours, total proteins were extracted from the cells using SDS lysis buffer (Beyotime, Shanghai, China, P0013G). The protein concentration was determined according to the instructions provided with the Bradford Protein Assay Kit (Detergent Compatible) (Beyotime, Shanghai, China, P0006C). Proteins were separated using SDS-PAGE Quick Gel Preparation Kit (Beyotime, Shanghai, China, P0012AC) and then transferred onto a PVDF membrane (Beyotime, Shanghai, China, FFP24). The membrane was blocked using QuickBlock™ Western Blocking Buffer (Beyotime, Shanghai, China, P0252-100ml). The primary antibodies used for incubation were as follows: MMP2 (Invitrogen, USA, PA5-85197, Rabbit, 1:2000 dilution), MMP9 (Invitrogen, USA, MA5-32705, Rabbit, 1:1000 dilution), VEGFA (Invitrogen, USA, MA5-13182, Mouse, 1:100 dilution), IGF1R (Invitrogen, USA, PA5-79444, Rabbit, 1:3000 dilution), FOXO1 (Invitrogen, USA, MA5-32114, Rabbit, 1:2000 dilution), p-FOXO1 Ser249 (Invitrogen, USA, PA5-105996, Rabbit, 1:2000 dilution), FOXO6 (Invitrogen, USA, PA5-106411, Rabbit, 1:2000 dilution), and GAPDH (Beyotime, Shanghai, China, AF1186, Rabbit, 1:3000 dilution) as the internal control. The membranes were then incubated with horseradish peroxidase (HRP)-conjugated goat anti-mouse IgG(H+L) (Beyotime, Shanghai, China, A0216, 1:1000 dilution) or HRP-conjugated goat anti-rabbit IgG(H+L) (Beyotime, Shanghai, China, A0208, 1:1000 dilution) as secondary antibodies. Protein bands were visualized using a SmartChemi III imaging system (Sage Creation, Beijing, China).

### 2.5 Statistical analysis

The statistical analysis of the data was conducted using SPSS 25.0 software. The data that followed a normal distribution were examined using either an independent sample t-test or a one-way analysis of variance. The data that did not follow a normal distribution were analyzed using a non-parametric test. The results were presented as the mean plus or minus the standard deviation (x ± s). A significance level of *P* < 0.05 was deemed statistically significant, whereas a significance level of *P* < 0.01 was also regarded statistically significant.

## 3 Results

### 3.1 Virtual screening predicts that HG exerts anti-invasive effects through the FOXO pathway

**Fig 1:**
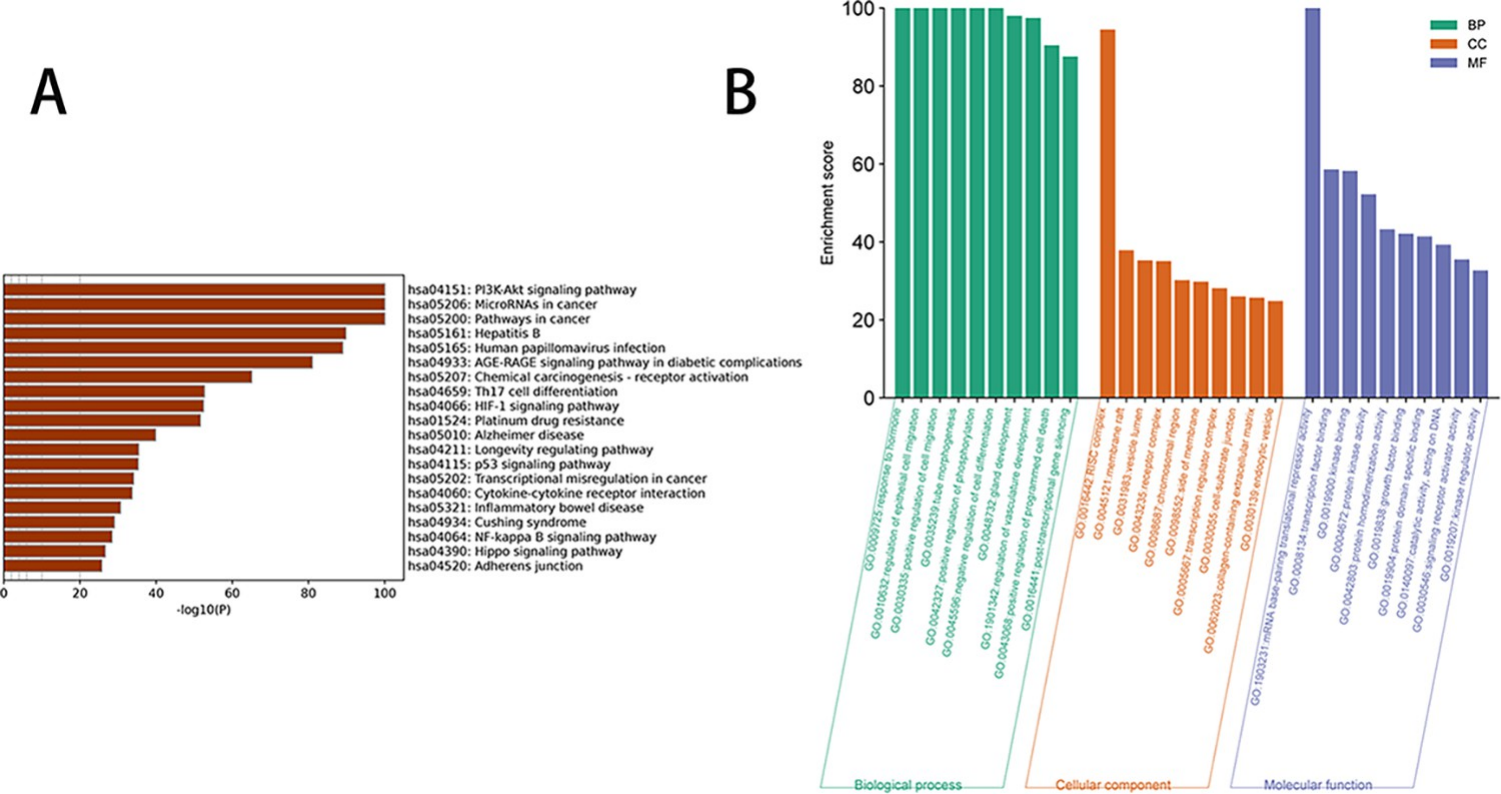
Enrichment analysis. (A) Liver cancer-related target enrichment analysis. (B) BP, CC and MF prediction analysis.

In this model, a total of 943 possible targets were ultimately obtained. The KEGG enrichment analysis reveals a high link between the invasion of liver cancer and the PI3K / AKT signaling pathway. Additionally, the mitogen-activated protein kinase (MAPK) pathway demonstrated a strong association in the further enhancement of the PI3K/AKT pathway, as depicted in Fig 1. Prior research has demonstrated that HG has the ability to control the PI3K/AKT [12], JAK/STAT [15], MAPK [22], and other pathways. These pathways serve as the shared precursor to the FOXO pathway. Hence, the subsequent course of action will involve doing a virtual screening of the FOXO signaling pathway.

**Fig 2:**
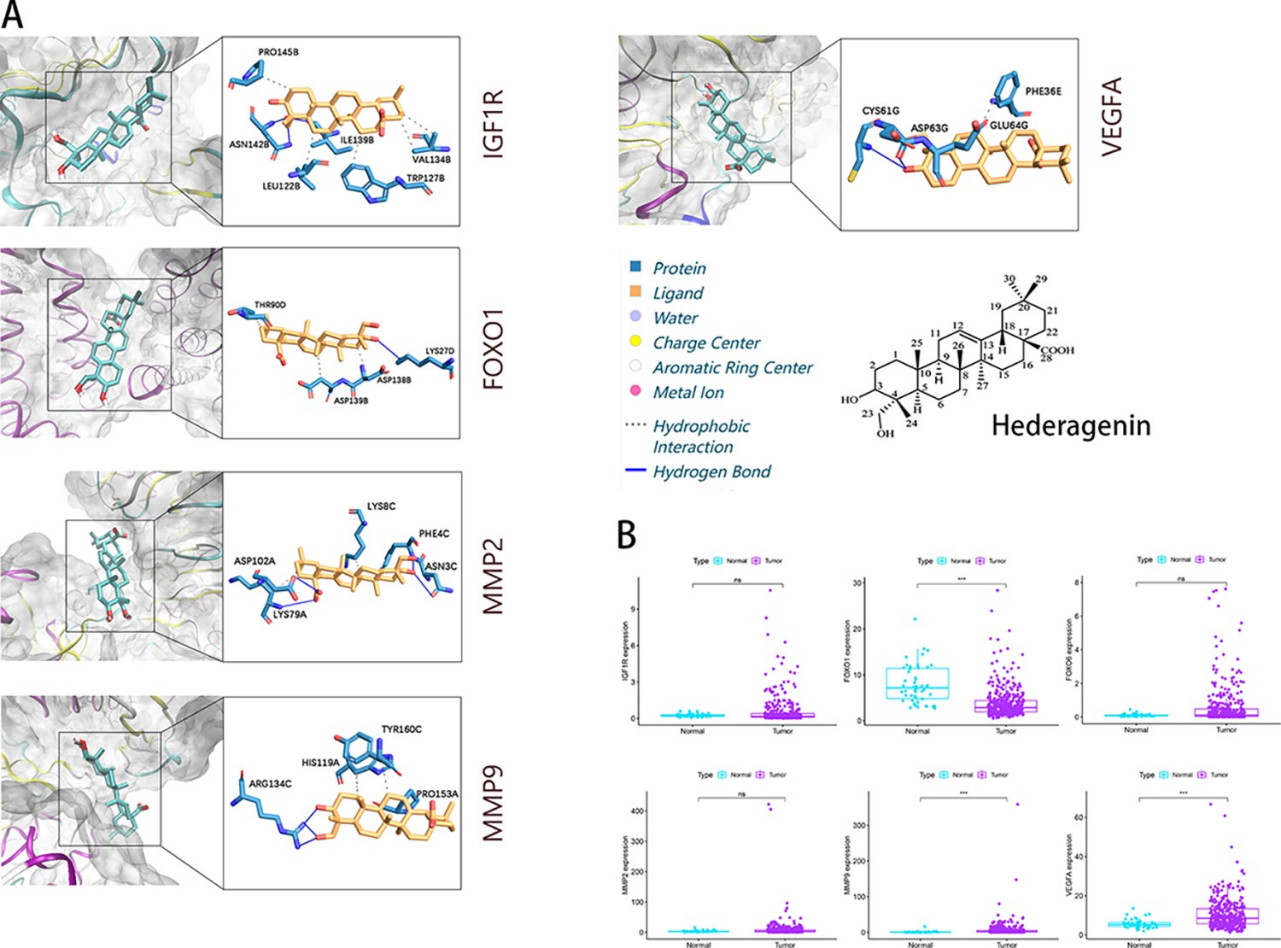
Molecular docking results. (A) Visualization and analysis of molecular docking. (B) Comparison of target protein expression in Normal and Tumor.

The expression levels of IGF1R, FOXO1, and FOXO6 were compared between Normal and Tumor samples using data from the TCGA database. Additionally, the expression levels of MMP2, MMP9, and VEGFA, which are known to promote invasion, were also compared (Fig 2B). The findings demonstrated a considerable inhibition of FOXO1 in the tumor (*P* < 0.001), while MMP9 and VEGFA were greatly enhanced in the tumor (*P* < 0.001). While there was no significant statistical difference in the expression of FOXO6 between the normal and tumor samples (*P* > 0.001), it was observed that FOXO6 was typically up-regulated in the tumor samples. By employing the molecular docking technique, the compound HG was sequentially docked with each expected target in order to determine the binding free energy. The results of this analysis can be found in Table 2. The Visual Molecular Dynamics software was employed to depict the ideal data for each docking group, while the Protein-Ligand Interaction Profiler [23] was utilized to examine the docking outcomes (Fig 2A).

**Table 2:** Target protein numbers and docking results.

Target protein	PDB number	Docking score (kcal·mol-1)
IGF1R	7XLC	-8.9
FOXO1	6QZR	-7.2
MMP2	7XGJ	-8.4
MMP9	5TH6	-8
VEGFA	3P9W	-7.8

### 3.2 The effect of HG on the activity of hepatocellular carcinoma cells

Based on the CCK-8 assay results assessing the impact of HG on the viability of Huh-7 and SMMC-7721 cells, the appropriate drug doses for the experiments were determined. The results indicated that HG inhibited the proliferation of both Huh-7 and SMMC-7721 cells in a dose-dependent manner (Fig 4C and 4D). The IC50 value for SMMC-7721 cells was found to be 72 μg/ml, while for Huh-7 cells, it was 57 μg/ml. Consequently, 20 μg/ml and 40 μg/ml were selected as the low and high doses for the experiments, respectively. These findings suggest that the inhibitory effect of HG on SMMC-7721 liver cancer cells is related to the drug concentration.

### 3.3 Effects of HG on apoptosis and necrosis of hepatocellular carcinoma cells

**Fig 3:**
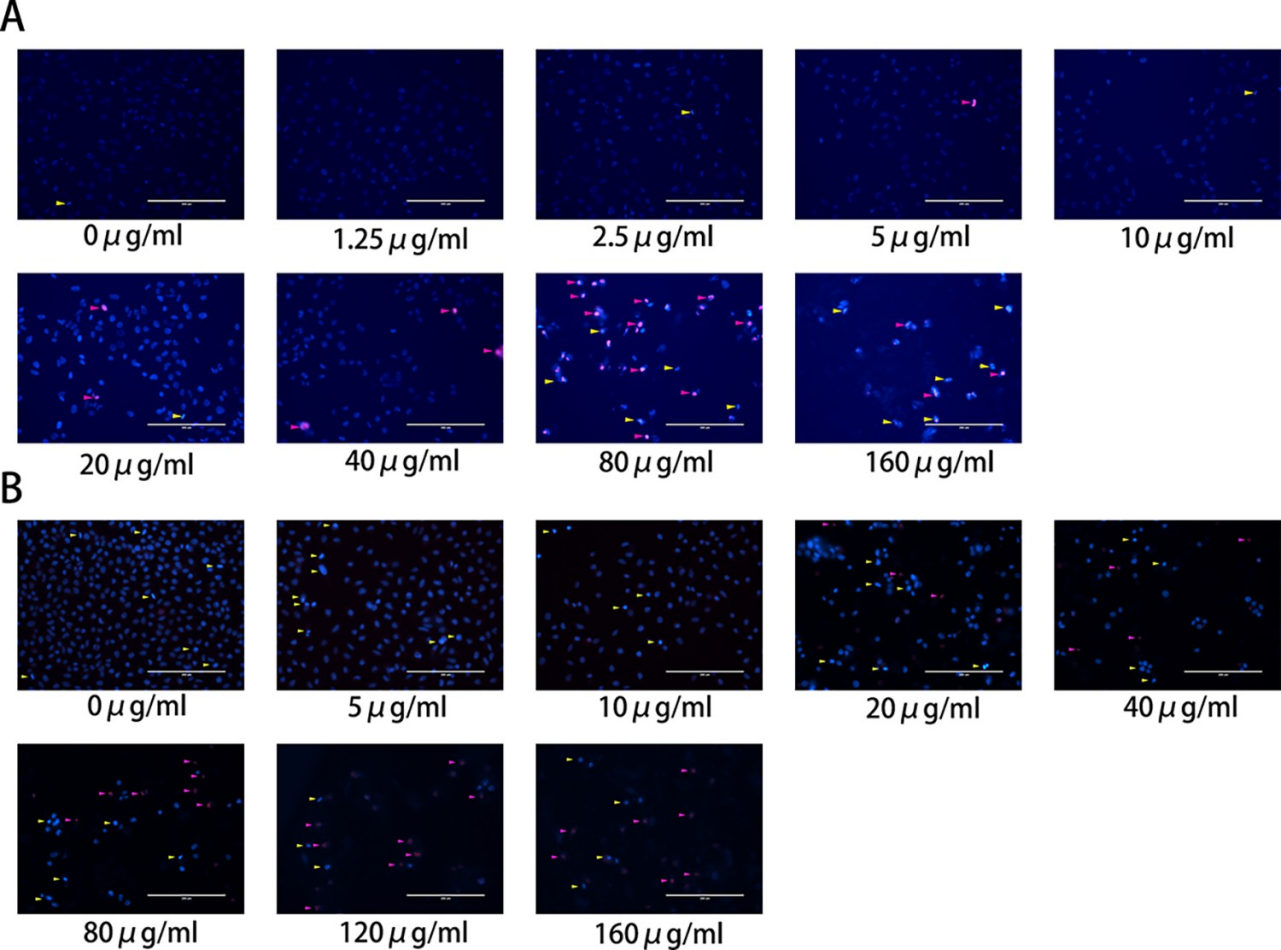
Fluorescence images of morphology by Hoechst 33342/PI double staining. (A) Assessment of apoptosis and necrosis in Huh-7 cells using Hoechst 33342/PI double staining at concentrations of 0, 1.25, 2.5, 5, 10, 20, 40, 80, and 160 μg/ml (yellow arrows indicate early apoptosis, pink arrows indicate necrosis/late apoptosis; scale bar = 200 μm).(B) Assessment of apoptosis and necrosis in SMMC-7721 cells using Hoechst 33342/PI double staining at concentrations of 0, 5, 10, 20, 40, 80, 160, and 320 μg/ml (yellow arrows indicate early apoptosis, pink arrows indicate necrosis/late apoptosis; scale bar = 200 μm).

Liver cancer cells were stained using Hoechst 33342 and propidium iodide (PI) solutions. The experiment demonstrated that, compared to the control group, the degree of apoptosis and necrosis gradually increased with higher concentrations of HG. At a concentration of 80 μg/ml, the number of necrotic cells exceeded the number of apoptotic cells (Fig 3A). These findings suggest that the antitumor effect of HG may be achieved by inducing apoptosis and necrosis in liver cancer cells.

### 3.4 HG can significantly inhibit the metastasis of liver cancer cells

**Fig 4:**
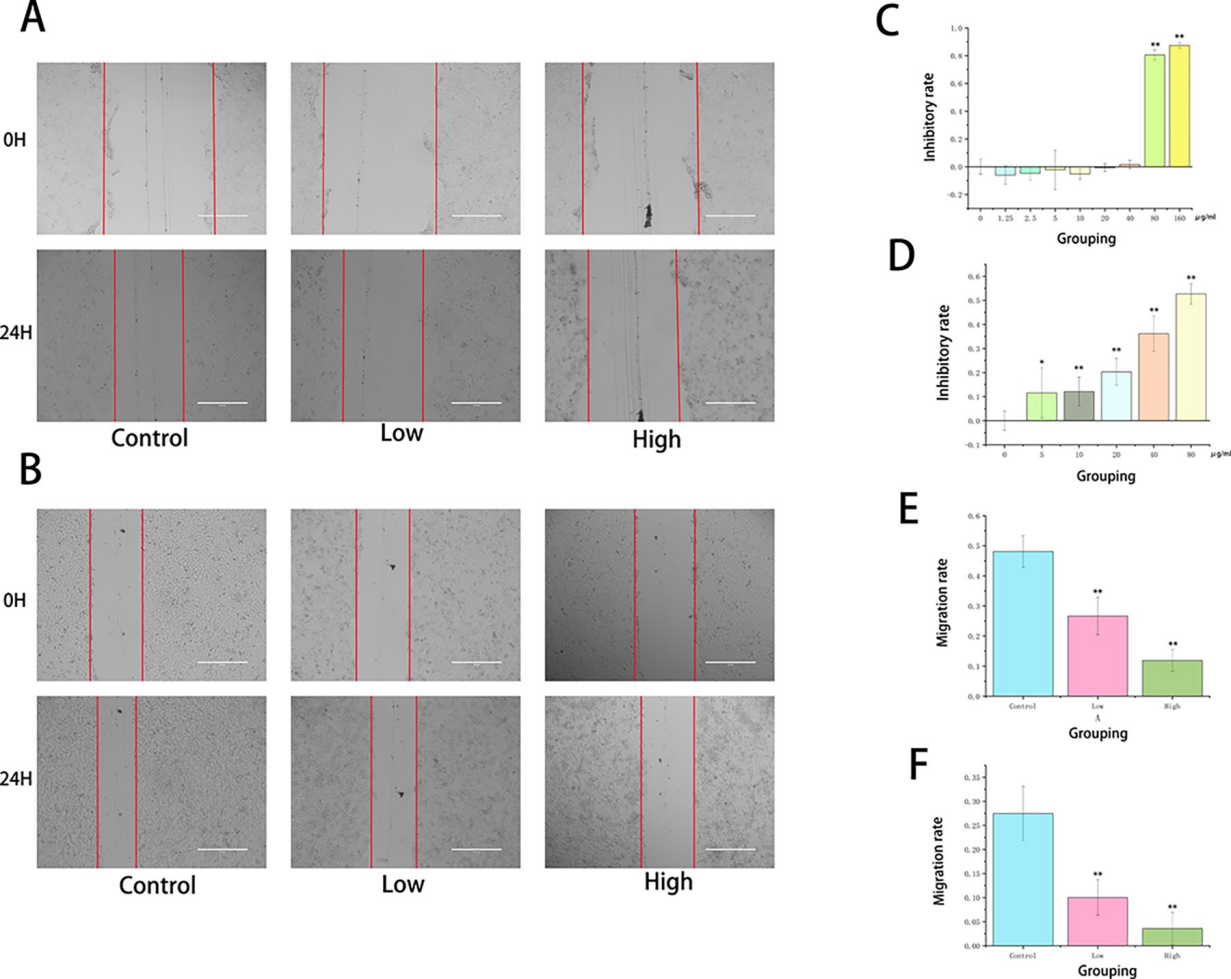
The effects of HG on the migration and proliferation. (A) Evaluation of Huh-7 cell migration using the scratch assay at different drug concentrations (scale bar = 400 μm). (B) Evaluation of SMMC-7721 cell migration using the scratch assay at different drug concentrations (scale bar = 400 μm). (C) Assessment of Huh-7 cell viability using the CCK-8 assay at concentrations of 0, 1.25, 2.5, 5, 10, 20, 40, 80, and 160 μg/ml (n = 6 after removing outliers; *: *P* < 0.05, **: *P* < 0.01 compared with the control group). (D) Assessment of SMMC-7721 cell viability using the CCK-8 assay at concentrations of 0, 5, 10, 20, 40, 80, 160, and 320 μg/ml (n = 6 after removing outliers; *: *P* < 0.05, **: *P* < 0.01 compared with the control group). (E) Migration rate of Huh-7 cells in each group from the scratch assay (compared with the control group, *: *P* < 0.05, **: *P* < 0.01). (F) Migration rate of SMMC-7721 cells in each group from the scratch assay (compared with the control group, *: *P* < 0.05, **: *P* < 0.01).

**Fig 5:**
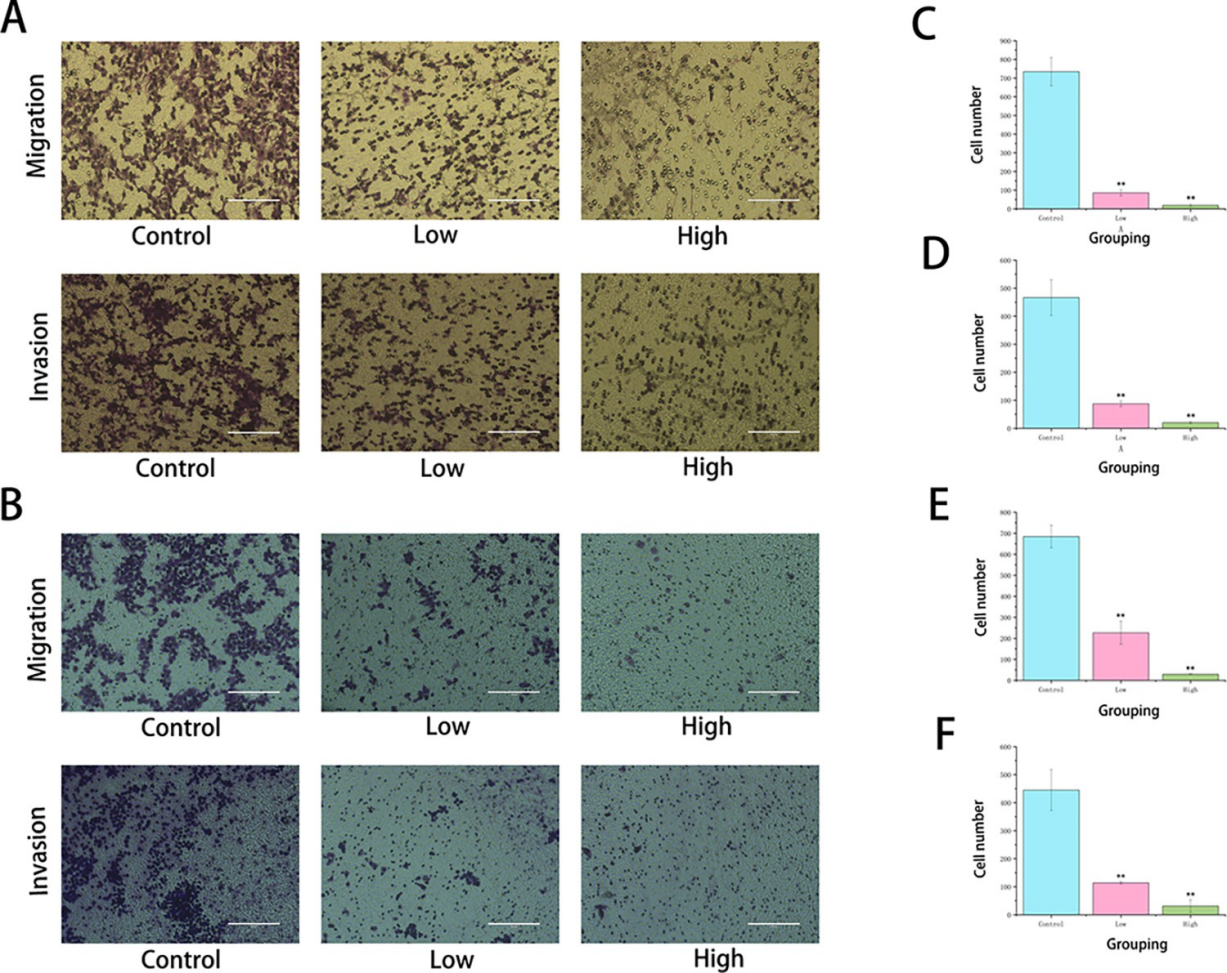
Transwell migration assay. (A) Evaluation of Huh-7 cell migration and invasion using the Transwell assay with or without Matrigel at specific drug concentrations (scale bar = 400 μm). (B) Evaluation of SMMC-7721 cell migration and invasion using the Transwell assay with or without Matrigel at specific drug concentrations (scale bar = 400 μm). (C, D) Transmembrane migration and invasion of Huh-7 cells in each group (compared with the control group, **: *P* < 0.01). (E, F) Transmembrane migration and invasion of SMMC-7721 cells in each group (compared with the control group, **: *P* < 0.01).

To verify the effects of HG on the migration and invasion of liver cancer cells, a combination of scratch and Transwell assays was used. In the scratch assay, comparing the scratch area at 0h and 24h (Fig 4A and 4B) revealed that the migration rate decreased as the drug concentration increased. At low doses, HG significantly inhibited the migration ability of liver cancer cells, indicating that HG can suppress cell migration and invasion in a dose-dependent manner (Fig 4E and 4F). The Transwell assay (Fig 5A and 5B) further confirmed that HG inhibits liver cancer cell migration and invasion in a dose-dependent fashion. Compared to the control group, both the low-dose group without Matrigel and the low-dose group with Matrigel showed a consistent reduction in the number of transmembrane cells, indicating decreased invasive capacity correlated with the drug concentration (Fig 5C and 5F). In summary, HG was found to inhibit liver cancer cell migration and invasion in a dose-dependent manner.

### 3.5 Effect of HG on the monoclonal ability of hepatocellular carcinoma cells

**Fig 6:**
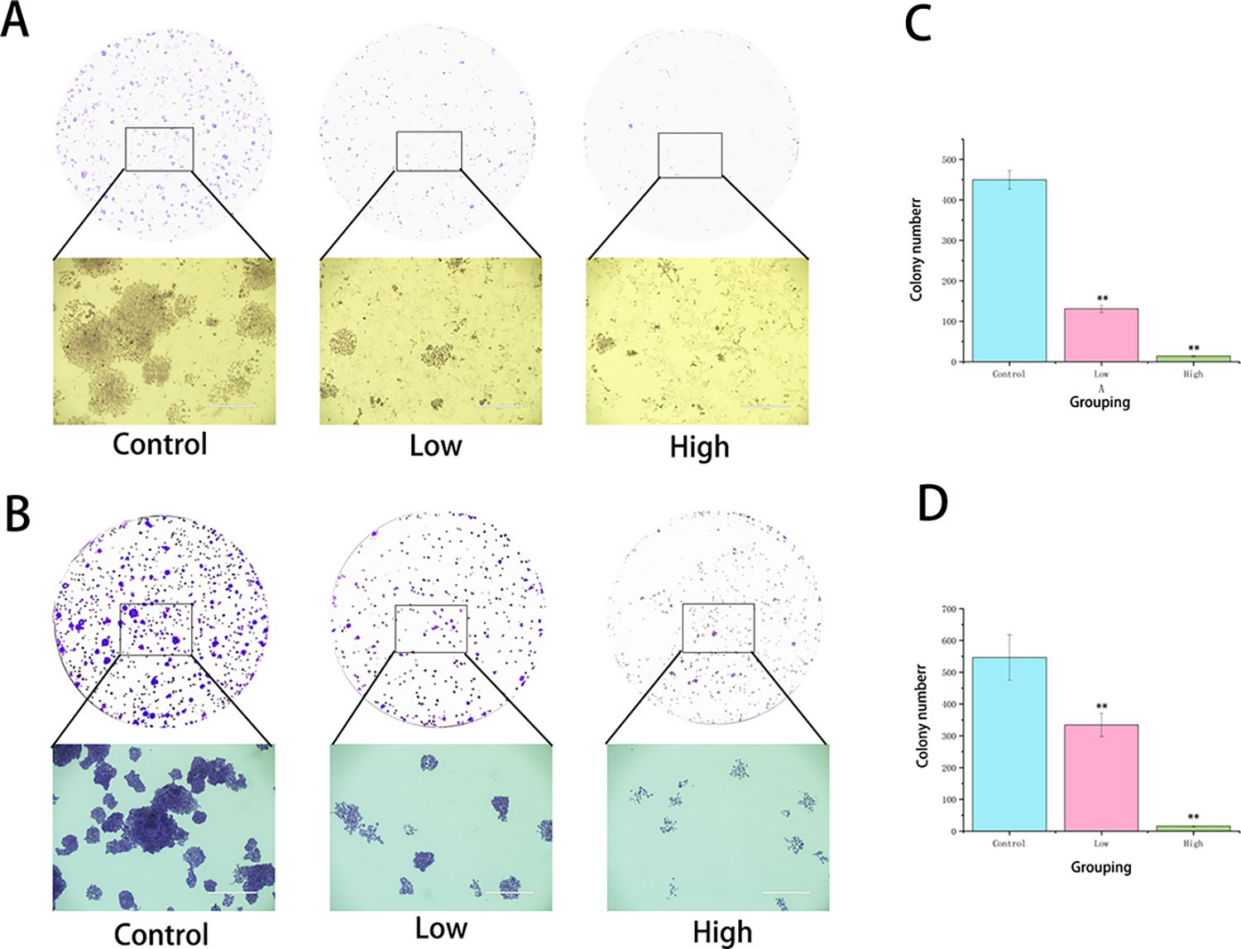
The effect of HG on cell cloning ability. (A Evaluation of Huh-7 single-cell cloning ability under different drug concentrations using a colony formation assay (n = 3, scale bar = 1000 μm). (B) Evaluation of SMMC-7721 single-cell cloning ability under different drug concentrations using a colony formation assay (n = 3, scale bar = 1000 μm). (C) Number of colonies formed in Huh-7 cells across different groups (compared with the control group, **: *P* < 0.01). (D) Number of colonies formed in SMMC-7721 cells across different groups (compared with the control group, **: *P* < 0.01).

The impact of HG on the clonogenic ability of liver cancer cells was assessed using a plate colony formation assay. The results showed that, compared to the control group, there was no significant cell death in the treatment groups; however, the clonogenic ability of the cells was markedly inhibited in a dose-dependent manner (Fig 6A and 6D). Therefore, HG can affect the clonogenic ability of liver cancer cells in a dose-dependent manner.

### 3.6 HG exerts anti-tumor effects by affecting the expression of FOXO1 and FOXO6

**Fig 7:**
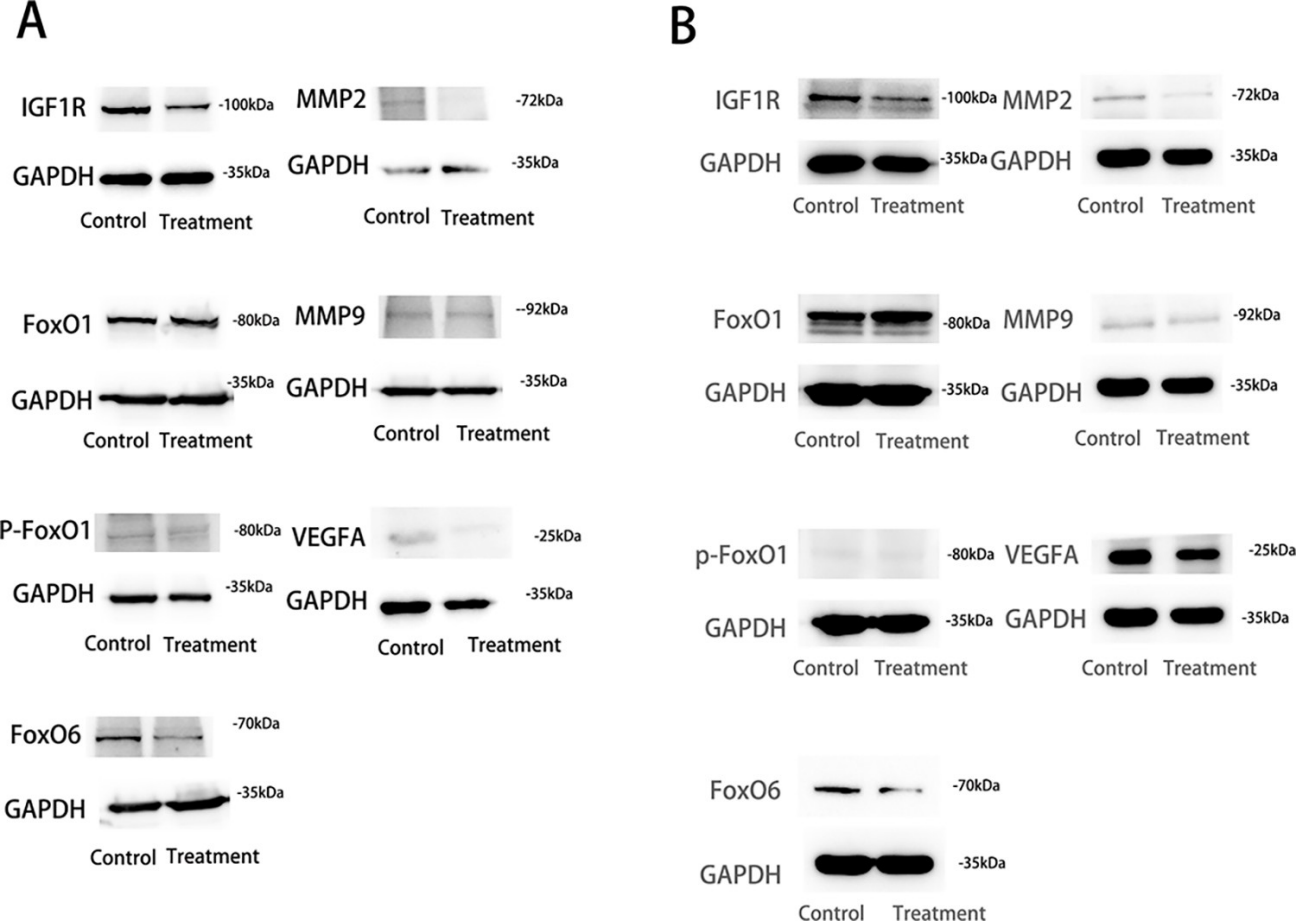
FOXOs and migration-related protein expressio. (A) Protein expression levels of FOXO1, FOXO6, IGF1R, p-FOXO1 Ser249, MMP2, MMP9, and VEGFA in Huh-7 cells for the control and treatment groups (n = 3, compared with the control group, *: *P* < 0.05).(B) Protein expression levels of FOXO1, FOXO6, IGF1R, p-FOXO1 Ser249, MMP2, MMP9, and VEGFA in SMMC-7721 cells for the control and treatment groups (n = 3, compared with the control group, *: *P* < 0.05).

**Fig 8:**
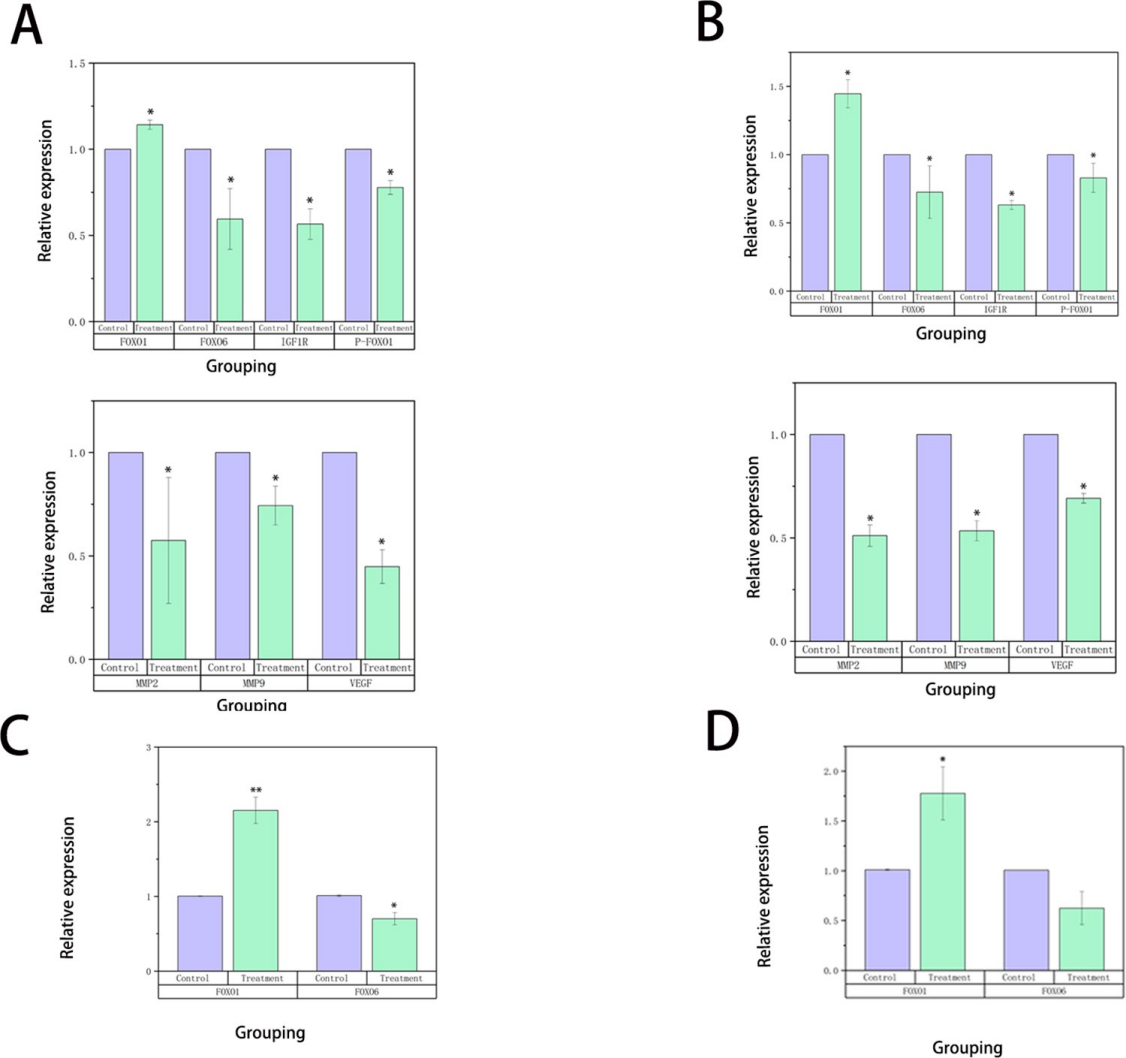
The analysis of migration-related gene and protein expression. (A) Protein expression levels of FOXO1, FOXO6, IGF1R, p-FOXO1 Ser249, MMP2, MMP9, and VEGFA in Huh-7 cells for the control and treatment groups (n = 3, compared with the control group, *: *P* < 0.05). (B) Protein expression levels of FOXO1, FOXO6, IGF1R, p-FOXO1 Ser249, MMP2, MMP9, and VEGFA in SMMC-7721 cells for the control and treatment groups (n = 3, compared with the control group, *: *P* < 0.05). (C) qRT-PCR analysis comparing the mRNA expression levels of FOXO1 and FOXO6 between control and treatment groups in Huh-7 cells (n = 3, compared with the control group, *: *P* < 0.05). (D) qRT-PCR analysis comparing the mRNA expression levels of FOXO1 and FOXO6 between control and treatment groups in SMMC-7721 cells (n = 3, compared with the control group, *: *P* < 0.05).

Whether HG exerts its antitumor effects by influencing FOXO expression through the regulation of the IGF1R pathway has not been previously studied. Therefore, our research group examined IGF1R expression. The results of the Western blot analysis (Fig 7A and 7B) demonstrated that HG inhibited the protein expression of IGF1R, FOXO6, and p-FOXO1 Ser249, while promoting the protein expression of FOXO1. Additionally, qRT-PCR results (Fig 8C and 8D) showed that HG suppressed FOXO6 mRNA expression (*P* < 0.05) and enhanced FOXO1 mRNA expression (*P* < 0.05). The trends in FOXO1/FOXO6 protein expression were consistent with the mRNA expression levels. These findings suggest that HG may exert its antitumor effects by regulating IGF1R, which in turn affects the phosphorylation of FOXO1 and the expression of FOXO6, ultimately inhibiting the migration and invasion of liver cancer cells.

### 3.7 HG inhibits the migration and invasion of HCC by affecting the expression of MMP2 / 9 and VEGFA

MMP2 and MMP9 play crucial roles in tumor invasion by degrading extracellular matrix proteins, thereby influencing tumor migration and invasion. VEGF, which is often overexpressed in various tumors, is associated with poor prognosis. The results of the Western blot analysis (Fig 8A and 8B) indicated that HG significantly inhibited the protein expression of MMP2 and MMP9 (*P* < 0.05) and downregulated the expression of VEGFA (*P* < 0.05). These findings suggest that HG can suppress tumor cell migration and invasion by inhibiting the expression of MMP2, MMP9, and VEGFA.

## 4.Discussion

Compared to the limited efficacy of surgical treatments for advanced-stage cancer patients, exploring the active components in natural anticancer medicinal plants in combination with anticancer therapies to enhance efficacy, reduce side effects, and mitigate resistance presents a novel therapeutic strategy. Inhibiting cancer cell migration and invasion is particularly crucial for advanced-stage patients. Therefore, we selected active compounds from natural plants to study their anticancer effects and mechanisms. Ivy, a natural anticancer plant, contains hederagenin (HG), which has been shown to possess extensive antitumor and anti-inflammatory activities [24]. The overexpression of inflammatory factors can create a pro-inflammatory environment within the body, fostering a microenvironment conducive to tumor cell growth, which is closely associated with poor cancer prognosis [25]. Previous studies have demonstrated that HG exerts anti-inflammatory effects by inhibiting the phosphorylation of the NF-κB signaling pathway, thereby downregulating the expression of inflammatory factors [26,27]. In terms of antitumor activity, our research found that HG dose-dependently inhibits the proliferation, migration, and invasion of liver cancer cells, suggesting that HG has the potential to be a targeted therapy for liver cancer.

The secretion of matrix metalloproteinases, particularly gelatinases like MMP2 and MMP9, is a critical step in tumor cell migration and invasion. MMP2 and MMP9 can degrade nearly all components of the extracellular matrix (ECM), enhancing the invasive ability of tumor cells. Overexpression of MMP2 and MMP9 can promote epithelial-mesenchymal transition (EMT), reduce cell adhesion, and accelerate tumor cell migration [28,29]. Additionally, tumor angiogenesis is regulated by the MMP family, with MMP2 and MMP9 being the primary proteases involved in tumor angiogenesis. MMP9 can increase the bioavailability of VEGF, thereby promoting tumor cell growth and migration. Therefore, reducing the expression of MMP2, MMP9, and VEGF is beneficial in inhibiting tumor cell migration and invasion, improving poor tumor prognosis, and increasing patient survival rates [30]. Our study demonstrated that HG significantly reduces the expression of MMP2, MMP9, and VEGF, thereby weakening the migration and invasion capabilities of liver cancer cells. The inhibition of tumor cell proliferation by HG was shown to occur through the induction of necrosis and apoptosis in tumor cells.

The FOXO family plays a vital role in cell proliferation, apoptosis, and migration. Dysregulation of FOXO function primarily occurs through various post-translational modifications, which can alter FOXO protein binding to DNA, affect subcellular localization, and thus modulate transcriptional activity. When IGF1 binds to IGF1R, it activates the downstream PI3K/AKT pathway, leading to the phosphorylation of FOXO proteins by p-AKT, causing nuclear-cytoplasmic shuttling. FOXO1 has phosphorylation sites at Thr24, Ser256, and Ser319 that can be modified by AKT. Phosphorylation of Ser256 inhibits FOXO1’s ability to bind DNA, while phosphorylation of Thr24 and Ser319 allows binding to 14-3-3 proteins, promoting nuclear exclusion and reducing transcriptional activity. Phosphorylated FOXO1 can be sequestered in the cytoplasm and targeted for degradation via the ubiquitin-proteasome system [31]. Studies have shown that FOXO1 overexpression has a positive effect on inhibiting tumor migration and proliferation, limiting EMT, and suppressing angiogenesis. Silencing FOXO1 increases EMT expression, ultimately promoting tumor cell migration [32].

FOXO6, when phosphorylated by AKT, has only two phosphorylation sites (Thr26, Ser184). Due to the lack of a nuclear export sequence, FOXO6 remains localized in the nucleus, unaffected by nuclear-cytoplasmic shuttling. Previous studies have shown that FOXO6 overexpression is associated with poor tumor prognosis. Research by Wang et al. demonstrated that FOXO6 overexpression increases tumor cell invasiveness and promotes malignant tumor progression [33]. Similarly, Lallemand et al. found that FOXO6 is closely associated with tumor progression and poor prognosis, with FOXO6 overexpression promoting cell proliferation, potentially linked to the suppression of the PI3K/AKT signaling pathway [34]. The underlying mechanisms require further investigation. In this study, we found that HG inhibits tumor cell migration and proliferation and delays malignant tumor progression by upregulating FOXO1 and downregulating the expression of IGF1R, FOXO6, and p-FOXO1.

## 5 Conclusion

In conclusion, our study is the first to demonstrate that HG regulates the phosphorylation of FOXO1, thereby affecting the proliferation, migration, and invasion of liver cancer cells. Our findings indicate that HG can inhibit the migration of liver cancer cells in vitro. The data suggest that the HG-mediated FOXO1/FOXO6 axis holds promise as a novel therapeutic target.

## Supporting information

S1 File Related experimental raw data of Huh-7. (XLSX)

S2 File Related experimental raw data of SMMC-7721. (XLSX)

S3 File Original images for blots and gels. (PDF)
