# Supplementary figures and images for: Hederagenin regulates the migration and invasion of hepatocellular carcinoma cells through FOXO signaling pathway
Source: PLoS One. 2024 Oct 9;19(10):e0310930. doi: 10.1371/journal.pone.0310930 (PMC11463763; doi:10.1371/journal.pone.0310930)

Fig.7A

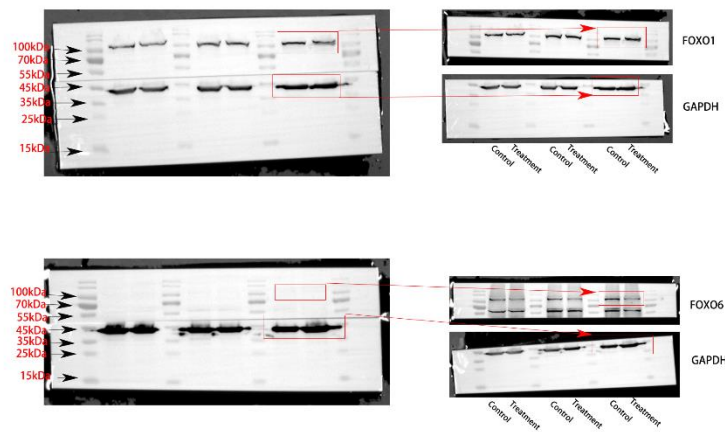

Fig.7A

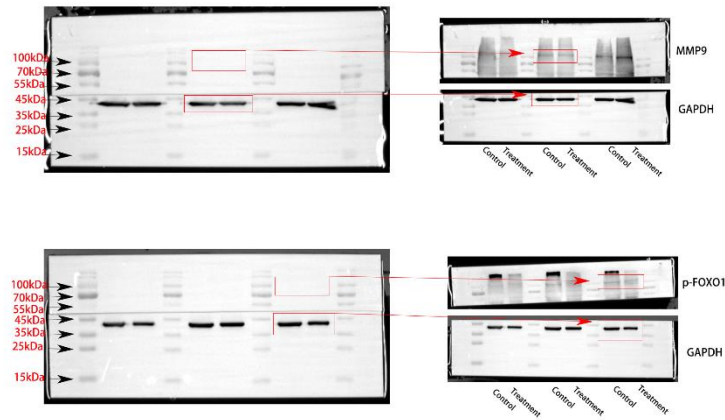

Fig.7A

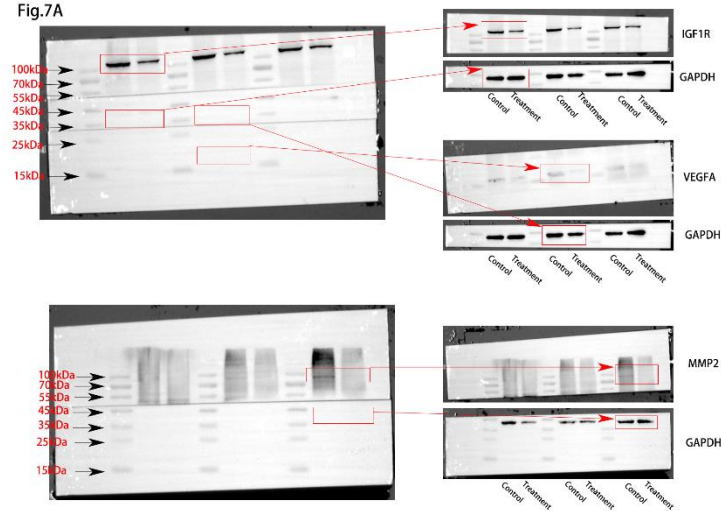

Fig.7B

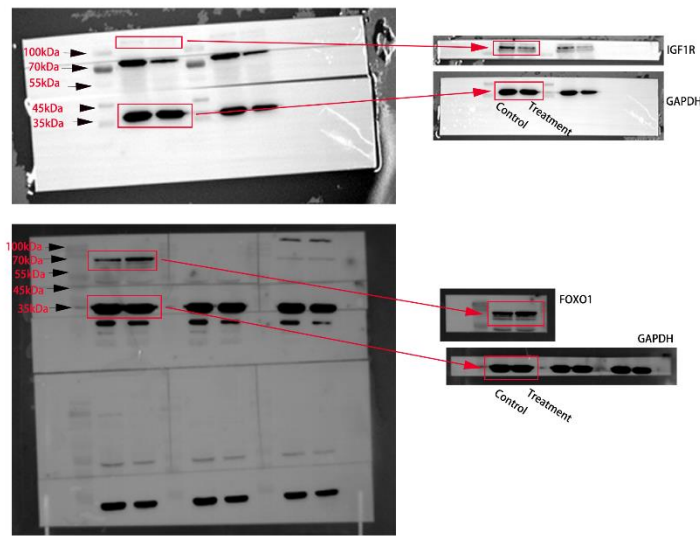

Fig.7B

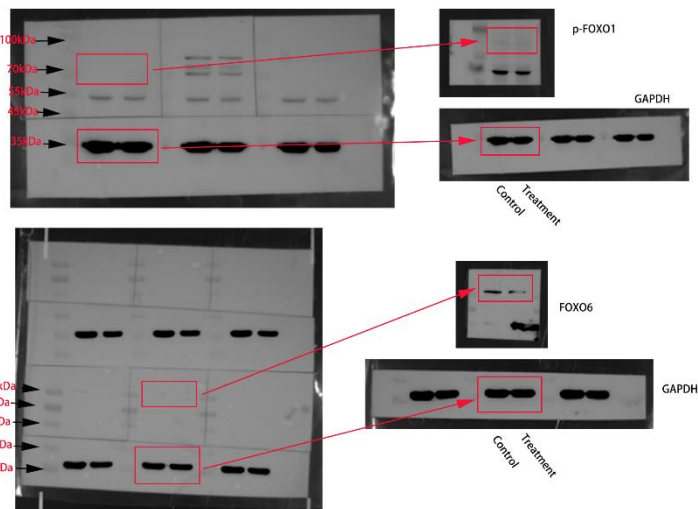

Fig.7B

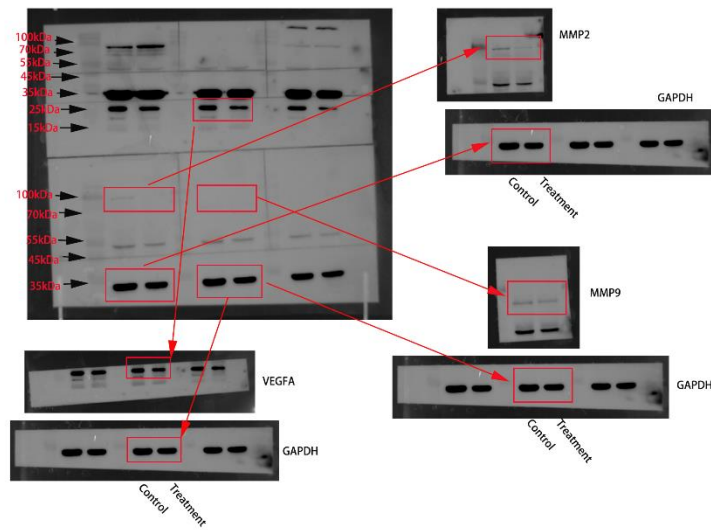

Supplement: S3 File — (PDF) [file pone.0310930.s003.pdf]
